# Adult diagnosis of Swyer-James-MacLeod syndrome: a case report

**DOI:** 10.1186/1752-1947-5-2

**Published:** 2011-01-04

**Authors:** Carlos Capela, Paulo Gouveia, Marco Sousa, Maria J Regadas

**Affiliations:** 1 Department of Internal Medicine, S. Marcos Hospital, Braga, Portugal; 2 School of Health Sciences, University of Minho, Braga, Portugal

## Abstract

**Introduction:**

Swyer-James-MacLeod syndrome or unilateral hyperlucent lung syndrome is a rare entity associated with postinfectious bronchiolitis obliterans occurring in childhood. It is characterized by hypoplasia and/or agenesis of the pulmonary arteries resulting in pulmonary parenchyma hypoperfusion.

**Case presentation:**

Here we report the case of a 53-year-old Caucasian woman with Swyer-James-MacLeod syndrome found in the differential diagnosis workup for a new onset of heart failure, secondary to pulmonary arterial hypertension complicated by a patent ductus arteriosus.

**Conclusion:**

Typically, this disorder is diagnosed in childhood after evaluation for recurrent respiratory infections, but sometimes an indolent course means diagnosis is not made until adulthood.

## Introduction

Swyer-James-MacLeod Syndrome (SJMS) or unilateral hyperlucent lung syndrome is a rare entity associated with postinfectious bronchiolitis obliterans occurring in childhood [1]. It is characterized by hypoplasia and/or agenesis of the pulmonary arteries resulting in pulmonary parenchyma hypoperfusion, showing a characteristic radiological pattern, such as translucent or hyperlucent unilateral lung [2]. Typically, this disorder is diagnosed in childhood after an evaluation for recurrent respiratory infections but sometimes patients who have little or no sequelae bronchiectasis have minor symptoms or are asymptomatic and may, therefore, miss their diagnosis until adulthood [3]. Here we presented a 53-year-old woman with SJMS found in the differential diagnosis workup for a new onset of heart failure.

## Case presentation

A 53-year-old Caucasian woman presented to the emergency department with a one month history of progressive dyspnea on exertion, paroxysmal nocturnal dyspnea, general edema and central cyanosis.

Her medical history was notable for recurrent pulmonary infections in childhood and an unstudied chronic productive cough not related to tobacco use. She was not given any lifelong medical attendance. No other family or pharmacological relevant antecedents were known. On physical examination she was not in distress. Her blood pressure was 124/67 mm Hg, pulse rate of 76 beats per minute, respiratory rate of 20 breaths per minute with an oxygen saturation of 85% while breathing room air. Her lungs had bibasilar crackles on auscultation. A 3/6 holosystolic murmur was present in the aortic area with back radiation. An elevated jugular venous pressure was noted and she presented with mild bilateral edema of the legs. Mild cyanosis of the lips and nail beds was also evident. The remainder of her physical examination was normal.

**Figure 1:**
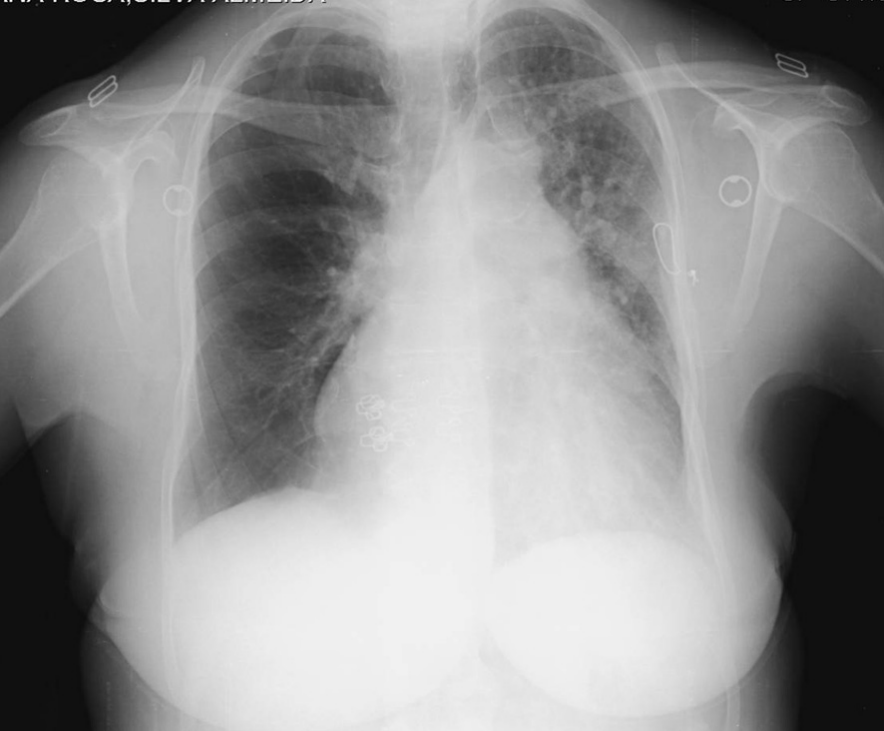
**Chest X-ray on admission to the emergency department**.

She had a white-cell count of 7600 per mm^3^, a hematocrit value of 57% and her platelet count was 108,000 per mm^3^. Levels of serum electrolytes, creatinine and urea nitrogen were normal and levels of hepatic transaminases, serum alkaline phosphatase, bilirubin, total protein and albumin were also normal. Cardiac enzymes were negative and the level of B-type natriuretic peptide was 230 pg per mL (normal range 0 to 100). Arterial blood gas values obtained while the patient was breathing room air revealed pH of 7.36, a carbon dioxide partial pressure of 56 mmHg, an oxygen partial pressure of 60 mm Hg, a bicarbonate level of 32 mmol per liter and an oxygen saturation of 87%. Furthermore, with supplemented oxygen of 24% and 28% the oxygen partial pressure was raised to 62 mm and 65 mm Hg, respectively. An electrocardiogram showed sinus rhythm, a 'strain' pattern on anterior leads (< 1 mm) and normal axis. A chest X-ray (Figure 1) showed a mild enlarged cardiac silhouette and what was first described as an alveolar-interstitial congestion pattern on the left side. A subsequent transthoracic echocardiogram (TTE) with Doppler showed normal sized cardiac chambers, a conserved left ventricular ejection fraction. A moderate, almost continuous but predominantly diastolic, color flow in the main pulmonary artery distal to the pulmonary valve was present and compatible with a patent ductus arteriosus (PDA) with minimal right-to-left shunt associated with an estimated pulmonary arterial pressure of 55 mmHg.

**Figure 2:**
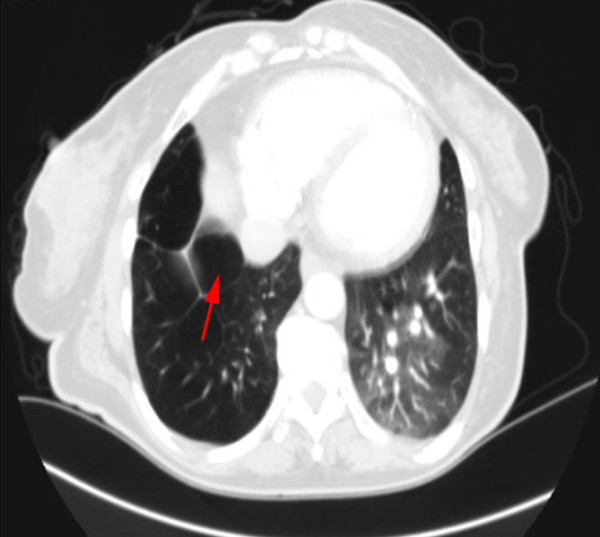
**Chest high resolution computed tomography scan demonstrating hyperinflation (air trapping) in the pulmonary parenchyma (red arrow)**.

**Figure 3:**
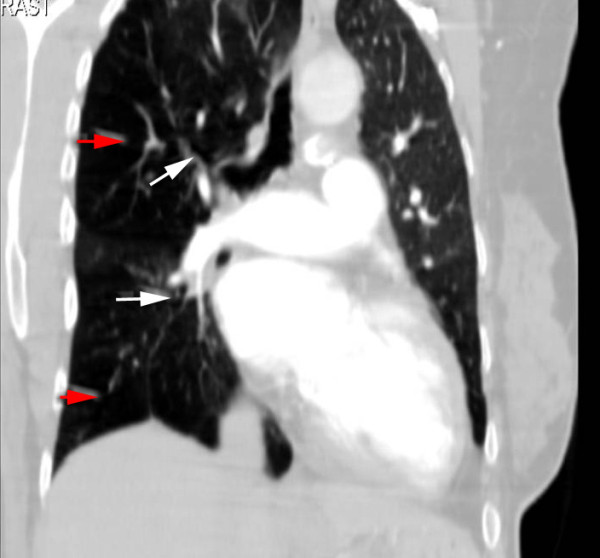
**Chest angio-computed tomography revealing hypoplasia/agenesis of the right pulmonary artery (white arrows) and hyperinflation of the pulmonary parenchyma (red arrows)**.

The patient was admitted to the internal medicine department for further evaluation. She was initially managed with furosemide (20 mg three times a day) and supplemented oxygen with a 24% face mask. A therapeutic phlebotomy of one unit blood (500 mL) was performed resulting with significant symptoms improvement. A pulmonary test function (PTF) revealed a relevant obstructive-restrictive pattern. A chest high-resolution computed tomography (HRCT) scan revealed hyperlucency and diminished vascularity in the right lower and middle lobe with hyperinflation of the pulmonary parenchyma (Figure 2) which was confirmed by the computed tomography (CT) angiography (Angio-CT) to be a diffuse hypoplasia/agenesis of right superior and inferior branches of the pulmonary artery (Figure 3), compatible with SJMS. The patient declined cardiac catheterization and was ultimately oriented to a cardiothoracic surgery consultation. She was discharged on day ten maintaining the bronchodilator and heart failure therapeutics and influenza and pneumococcal vaccinations were recommended.

## Discussion

In this case, a multi-factorial heart failure was considered. The first impression from the radiological pattern was consistent with predominant pulmonary left-side edema related to heart failure. Finally, a radiological hypertransluced pattern on HRCT in the opposite lung was seen to be related to a diminished vascularity and hyperinflation pulmonary parenchyma, characteristically associated with SJMS. The relevant polycythemia was most probably secondary to cyanotic congenital heart disease associated with the newly diagnosed PDA with minimal right-to-left shunt. A PDA rarely closes spontaneously after infancy [4] and small patent ductus arteriosus cause no symptoms, as it seems in the present case. It is not clear which factor most contributed to the actual clinical picture presented by the patient: the PDA with a minimal inverted shunt; the parenchyma abnormalities resulting in differential pulmonary blood flow due to SJMS; or even the respiratory obstructive lung disease component seen on PTF. A cardiac catheterization would eventually be necessary but, at this point, she declined further evaluation. Nevertheless, the lack of normalization in arterial hypoxia in the presence of oxygen supplementation most probably shows a non-invasive confirmation of the major contribution of inverted shunt PDA which may explain the development of pulmonary arterial hypertension and consequent heart failure. The normal size right cardiac chambers shown in TTE should be better characterized with trans-esophageal echocardiography which was also declined by the patient.

SJMS is considered to be a relatively uncommon and complex disease characterized by unilateral hyperlucency of a part of or the entire lung which was first described in 1953 by Swyer and James [3]. It is presently considered to be an acquired disease secondary to viral bronchiolitis and pneumonitis in childhood etiological associated [1] with *Paramyxovirus morbillivirus*, *Bordetella pertussis*, *Mycobacterium tuberculosis*, *Mycoplasma pneumoniae*, influenza A and adenovirus types 3, 7 and 21. Our patient suffered recurrent episodes of pulmonary infections in her childhood. Clinically, patients usually present productive cough, shortness of breath and dyspnea on exertion, sometimes with haemoptysis. Some patients, who have little or no associated sequelae bronchiectasis, have minor symptoms or are asymptomatic and may, therefore, not be diagnosed until they are adults. In our case, the final SJMS diagnosis was reached during a new onset heart failure workup performed when the patent was 53. SJMS diagnosis is based on the radiological pattern such as [5] unilateral or lobar pulmonary hyperlucency associated with an air trapping lung during expiration ultimately resembling a mosaic pattern. The affected lung parenchyma shows a variable degree of destruction and bronchiectasis could be associated. Those aspects could better explain the obstructive-restrictive respiratory pattern typically seen on PTF and also reported in our patient. In addition, pathological pulmonary artery seen on angio-CT has, typically, a decreased caliber and, consequently, the lung blood flow is reduced. Finally, another characteristic described is that the pathological perfusion changes in the diseased lung sections and the occasionally bizarre hyperinflation ultimately result in the compression of healthy lung areas resulting in atelectasis [6]. For this reason, the diagnosis of this syndrome is better established with HRCT on inspiration and expiration complemented with an angio-CT. A ventilation-perfusion lung scanning [7] could also be performed but false-positives could appear in the presence of any disorder involving distal airway obstruction (such as, bronchiolitis obliterans, asthma or congenital lobar emphysema). SJMS treatment includes the early control of lung infections as well as influenza and pneumococcal vaccinations [2,5]. Resection of the affected lung was successful [2]. No specific morbid-mortality studies with SJMS have been done.

## Conclusion

In summary, the present case emphasizes that a chest X-ray may underestimate the prevalence of the SJMS syndrome. Despite numerous chest radiographic examinations, SJMS was not diagnosed until another complementary imaging study was performed. The main reason for our reporting this case was related to the fact that few cases have been reported worldwide of adults presenting with SJMS.

## Abbreviations

Angio-CT: angiography CT; CT: computed tomography; HRCT: high resolution CT; PDA: patent ductus arteriosus; PTF: pulmonary test function; SJMA: Swyer-James-MacLeod syndrome; TTE: transthoracic echocardiogram;

## Competing interests

The authors declare that they have no competing interests.

## Consent

Written informed consent was obtained from the patient for publication of this case report and any accompanying images. A copy of the written consent is available for review by the Editor-in-Chief of this journal.

## Authors' contributions

CC, PG and MS were responsible for the case review, literature review and the final drafting of the manuscript. MJR was responsible for the manuscript critique and review. All authors read and approved the final manuscript.
